# Anti-Oxidant, Anti-Inflammatory and Antiviral Properties of Luteolin Against SARS-CoV-2: Based on Network Pharmacology

**DOI:** 10.3390/ph18091329

**Published:** 2025-09-04

**Authors:** Xin Li, Yunmei Fu, Tong Yu, Ruizhe Song, Hongguang Nie, Yan Ding

**Affiliations:** 1 Department of Chemistry, School of Forensic Medicine, China Medical University, Shenyang 110122, China; 20071029@cmu.edu.cn; 2 Department of Stem Cells and Regenerative Medicine, College of Basic Medical Science, China Medical University, Shenyang 110122, China; ymfu@cmu.edu.cn (Y.F.); yutong@cmu.edu.cn (T.Y.); 2023120035@cmu.edu.cn (R.S.)

**Keywords:** luteolin, severe acute respiratory syndrome coronavirus 2, coronavirus disease 2019, network pharmacology, angiotensin-converting enzyme 2

## Abstract

Luteolin is a natural flavonoid compound with multifaceted pharmacological properties, including anti-oxidant, anti-inflammatory, antiviral, and anti-tumor activities. Network pharmacology analysis has been utilized to decipher the underlying mechanisms and multitargets of luteolin against coronavirus disease 2019 (COVID-19). This review aims to provide a systematic and comprehensive summary of luteolin, as a potential novel remedy with anti-severe acute respiratory syndrome coronavirus 2 (SARS-CoV-2) activity, as well as its anti-oxidant mechanisms. We systematically delineate the epidemiological profile, genomic architecture, and replicative dynamics of SARS-CoV-2, thereby constructing a multiscale framework to decode its pathogenic mechanisms. Employing a multi-level network pharmacology analytical strategy, we identify 46 core targets through protein interaction network construction, followed by Gene Ontology and Kyoto Encyclopedia of Genes and Genomes enrichment analysis. Molecular investigations reveal luteolin’s dual antiviral mechanisms, including direct targeting of SARS-CoV-2 proteins and host-directed intervention through suppression of angiotensin-converting enzyme 2 receptor engagement/transmembrane protease serine 2-mediated viral priming. The polypharmacological profile of luteolin demonstrates synergistic effects in blocking viral entry, replication, and host inflammatory cascades. This phytochemical repurposing study of luteolin provides a novel mechanistic paradigm for developing multitarget antiviral agents, highlighting the translational value of natural compounds in combating emerging viral variants.

## 1. Introduction

The epidemic coronavirus disease 2019 (COVID-19) results from the infection of severe acute respiratory syndrome coronavirus 2 (SARS-CoV-2), which shares 79.6% genomic homology with SARS-CoV [1]. COVID-19 has the potential to rapidly progress into mild to severe acute respiratory distress syndrome (ARDS), even multiple organ dysfunction or mortality [2,3,4]. As of 17 March 2025, the total number of COVID-19 patients worldwide has reached 2.2 million.

To date, approximately 85% natural products, including various plant/herbal crude extracts or fractions, have been extensively evaluated for their potential roles against COVID-19 [5]. Given the low toxicity and availability of certain active compounds, *Lonicera japonica* Thunb., commonly known as Jinyinhua in China, is identified as a promising candidate that inhibits the coronavirus–host protein pathways and disturbs various phases of the coronavirus life cycle, including virus invasion into the infected cells, replication, and assembly process [6]. Pharmacological studies have shown that Jinyinhua has a positive therapeutic effect on serious viral diseases, such as SARS and H1N1 virus [7]. At present, Jinyinhua Oral Liquid, as a Chinese patent medicine, has been registered in the clinical studies for COVID-19 [8].

**Figure 1:**
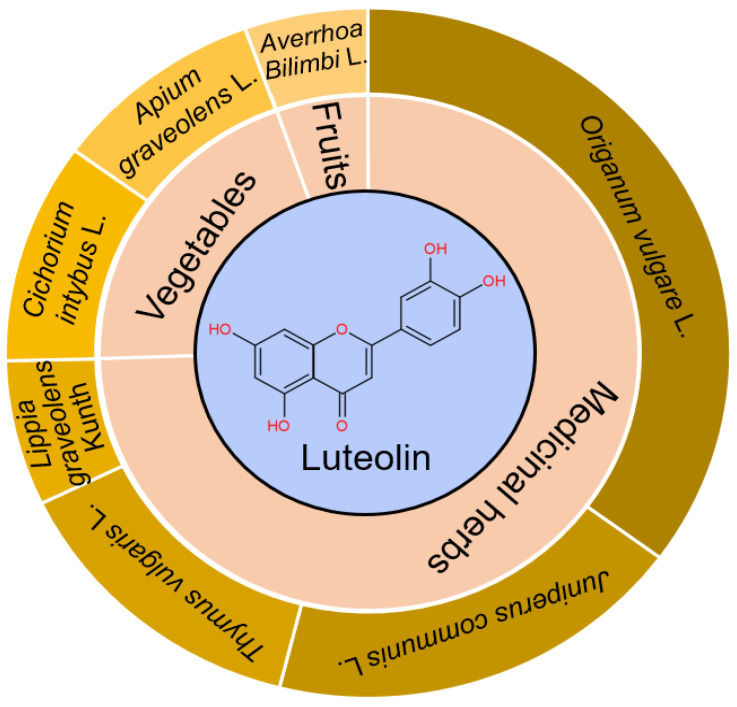
Major sources/structure of luteolin. Medicinal herbs, vegetables, and fruits containing the content of luteolin higher than 20 mg/100 g are summarized. Luteolin belongs to flavonoid with C6-C3-C6 as the parent nucleus, and has 4 phenolic hydroxyl groups in the site of C-3′, C-4′, C-5, and C-7, respectively.

As the main active constituents of *Lonicera japonica* Thunb. [9], luteolin (3′,4′,5,7-tetrahydroxyflavone, yellow needlelike crystals, CAS Registry Number: 491-70-3) and its derivatives, luteolin 7-O-α-D-glucoside and luteolin-7-O-β-D-galactoside, are derived from medicinal plants, fruits and vegetables [10,11]. Luteolin, a typical flavonoid, mainly exists in *Cichorium intybus* L., *Apium graveolens* L., *Averrhoa Bilimbi* L., *Origanum vulgare* L., *Juniperus communis* L., *Thymus vulgaris* L., and *Lippia graveolens* Kunth, with the content higher than 20 mg/100 g (Figure 1). Celery, broccoli, artichokes, green peppers, parsley, thyme, dandelion, perilla, chamomile tea, carrots, olive oil, peppermint, rosemary, navel oranges, and oregano are some examples of dietary sources containing luteolin [12].

The concentration of luteolin in plasma increases after consumption at 0.5 h and the peak level is at about 1.1 h after oral ingestion, measured by the high-performance liquid chromatography technique [13]. Thus, it has been suggested that luteolin is considerably absorbed after oral intake and seems to be quickly metabolized after absorption into other compounds [14]. The area under the concentration–time curves, apparent distribution volume, and renal clearance are 23.03 μgh/mL, 65.12 L/kg, and 8.473 L/kg, respectively. Following absorption, luteolin exhibits a tissue-specific distribution (liver > spleen > lung > kidney > heart) with hepatic generation of its primary metabolites, luteolin glucuronide and luteolin sulfate via glycosylation and sulfation [15], where the glucuronide conjugate is intracellularly converted to bioactive luteolin aglycone, suppressing inflammatory gene expression in LPS-stimulated RAW264.7 macrophages [16]. The key physicochemical properties of luteolin are shown in Table 1, which exhibits multifaceted biological benefits, such as anti-oxidant, anti-inflammatory, antiviral properties, anti-cancer, immune-regulatory, as well as cardio- and neuro-protective effects [17,18,19,20]. Emerging studies reveal that luteolin phytosomes effectively combat long-COVID-related brain fog primarily through a potent anti-inflammatory mechanism, i.e., suppressing mast cell-driven neuroinflammation [21]. The anti-oxidant effect of luteolin appears to work as a reactive oxygen species (ROS) scavenger [12]. It is demonstrated that luteolin can inhibit a range of viruses, including pseudorabies, respiratory syncytial virus, influenza A, dengue, Epstein–Barr, and Japanese encephalitis [22,23,24,25,26,27]. As far as SARS-CoV-2 is concerned, luteolin can effectively inhibit SARS-CoV-2 pseudovirus entrance into cells, possessing a low toxicity and wide range of potential dosages for clinical trials [28]. Flavonoids are categorized among the pan assay interference compounds (PAINs), and are suggested to obscure the results of various assays. Although identified as a PAIN, luteolin has been reported to inhibit virus infection on the premise of an inflammatory response occurring [29]. The biological activity including anti-oxidant, anti-inflammatory and antiviral properties of luteolin are highly necessary to discuss during SARS-CoV-2 infection. Network pharmacology is a valuable tool that describes the relationship among diseases, human biological systems, and drug targets based on biological system network analysis [30,31,32]. Precise and effective therapeutic intervention is achieved by synergistic network pharmacology and drug repurposing, obviating the need for drug discovery and speeding up clinical translation. Till now, it has become a popular trend to decipher the targets and clarify disease–gene relationships using network pharmacology, which can achieve precise drug intervention and accelerate clinical translation [33]. Through the molecular docking approach, luteolin is predicted to be the best inhibitor against S binding pocket angiotensin-converting enzyme 2 (ACE2). In this review, we integrate the interdisciplinary technologies of network pharmacology, providing the possible signaling pathways in the treatment of luteolin against COVID-19.

## 2. Luteolin Disrupts the Life Cycle of SARS-CoV-2 by Acting on Protein Assembly and RNA Synthesis

**Figure 2:**
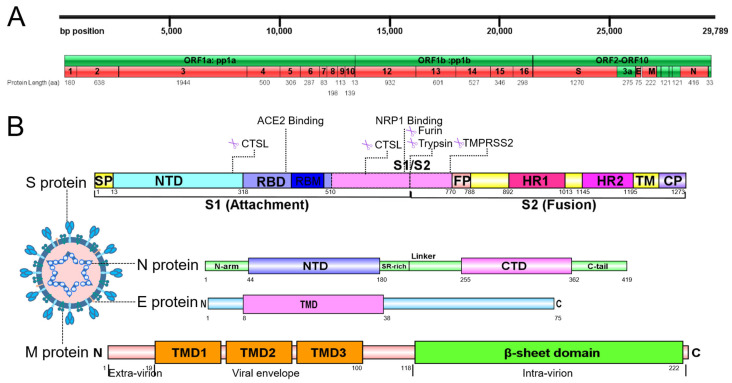
Overall genome and structural protein analysis of SARS-CoV-2. (**A**) Genome of SARS-CoV-2. (**B**) Structural domains of SARS-CoV-2 S protein and cleavage sites are highlighted. S protein comprises two regions: S1 with the receptor-binding domain (RBD) essential for the recognition of host receptor and S2, essential for membrane fusion and entry. Between S1 and S2 subunits, there is the polybasic sequence recognized by host endo-proteases furin. The activation site of S protein is recognized by serine protease TMPRSS2 in region S2′ of S2 domain. The S1 subunit includes the signal peptide (SP), N-terminal domain (NTD), and RBD, while fusion peptide (FP), heptad repeat 1/2 (HR1/2), transmembrane domain (TM), and cytoplasmic domain (CP) consist of S2 subunits. N protein contains 3 intrinsically unordered regions. N-arm, linker region and C-tail, and the NTD and C-terminal domain (CTD) of N protein are illustrated. The charged Ser/Arg (SR)-rich motif is shown. M protein consists of three structural segments. The N-terminal three transmembrane helices are mostly embedded in the viral envelope, and an inward-facing C terminal β-sheet sandwich domain are illustrated. E protein is a small 75-amino-acid integral membrane protein with one transmembrane domain, an intermediate helical domain, and hydrophilic N- and C-terminal domains.

Sequence analysis of SARS-CoV-2 extract embraces 29,891 nucleotides in size, encoding 9860 amino acids and 14 open reading frames (ORFs) [34,35]. The ORF1a/ORF1ab codes a polyprotein, which synthesizes 16 non-structural proteins (Nsps) that constitute the replicase/transcriptase complex (Figure 2A) [36,37]. At the 3’ terminal of viral genome, other ORFs code 4 structural proteins—S, nucleocapsid (N), membrane, and envelope—as well as 9 putative accessory factors, which are pivotal to viral replication and CoVs infection, as such are highly attractive targets for antiviral drug development of SARS-CoV-2 (Figure 2B) [38,39]. Viral proteins, including structural and non-structural, play critical roles in mediating host cell entry and replication processes, thereby representing promising therapeutic targets for antiviral development. Natural compounds targeting conserved viral domains exhibit therapeutic potential by multi-mechanistically disrupting viral entry, replication, and assembly, thereby addressing both drug resistance from protein mutations and off-target risks of synthetic inhibitors [40,41]. Intriguingly, luteolin can interfere in viral replication at an early stage of infection, and its binding energies to PL^pro^, 3CL^pro^, RdRp, and S protein were all more than −6.0 kcal/mol [33].

### 2.1. Disturbance of Virus Entry and Genome Packaging

Among the four main structural proteins SARS-CoV-2 genome encodes, the primary S protein is a homotrimeric glycoprotein, which is cleaved into S1 and S2 subunits by furin-like proteases during virus entry into the host [42]. Consequently, N protein participates in the package of virus RNA into SARS-CoV-2 particles during assembly [43]. Blocking the S protein to prevent virus entry is crucial in impeding early-stage viral propagation and mitigating drug resistance. Research demonstrates that luteolin interferes with the formation of the six-helical bundle fusion core by binding to specific residues in the receptor-binding domain (RBD), namely Asn343, Asp364, and Phe374, through hydrogen and π-H bonds. Meanwhile, luteolin can also form a hydrophobic interaction with Gln804 and Asn801 of S protein during COVID-19 [44,45]. Moreover, luteolin regulates cytokine levels by inhibiting JAK1/STAT3-related inflammation in S1-transfected alveolar epithelial cells [46].

Two independent folded domains named N-terminal domain (NTD) and CTD form N protein, linked with an inherently disordered Ser/Arg-rich connector (Figure 2B) [47]. Additionally, two deranged regions are located at the edges of NTD and CTD as N-arm and C-tail, respectively [48,49]. Besides the principle role to bind RNA genome of SARS-CoV-2 [50,51], N protein is also important for mRNA transcription/replication, cytoskeleton formation, and immunomodulation [48,52]. Luteolin can interact with RNA binding sites as well as predicted N protein interface with −7.5 kcal/mol binding energy, by structure-based molecular docking and all-atom molecular dynamics simulation approach. The step of RNA adhering to protein is interfered, after luteolin is connected to Asn76 of N-arm NTD protein by hydrogen bonds and Ser79, His146, Ile147, Trp53, Ala156, Ile158 through hydrophobic and Van der waal interactions [53].

### 2.2. Inhibition of Viral Enzymes and RNA Synthesis Machinery

The role of both proteases, PL^pro^ and 3CL^pro^, is to cleave polyprotein pp1a/1ab into nsp 1-16, which is indispensable to the processes for new virions to enter into infected cells, including transcription, translation, and replication [54,55,56]. PL^pro^ can also strip ubiquitin and ISG15 to facilitate SARS-CoV-2 moving away from the inherent immune system [57,58,59,60]. In addition, the substrate specificity of 3CL^pro^ is highly conserved among various CoVs [61,62]. The crucial replicative enzymes, including RdRp and helicase, are released by cleaving protein precursors for virus replication and packaging within host cells [63].

#### 2.2.1. PL^pro^ and 3CL^pro^

In ZINC database, about forty thousand natural product-like compounds are screened out, among which luteolin has inhibitory effects against 3CL^pro^ with IC_50_ value of 11.81 µM and the binding energy of −8.1 kcal/mol, respectively [31]. Meanwhile, by in silico screening of bioactive compounds isolated from *Rosmarinus officinalis* L., it is predicted that luteolin can bind 3CL^pro^ to treat SARS-CoV-2 infection with acceptable drug-likeness, pharmacokinetics, and ADMET characteristics [64]. The structure–activity relationship analysis of luteolin evidences that the C-3′ hydroxyl group in luteolin is evidenced to be important for the inhibition of SARS-CoV 3CL^pro^ [65]. Research from molecular docking further demonstrates that luteolin possesses lower binding energy by forming hydrophobic interaction and hydrogen bonds with Met49/Val3 and Gln189/Leu4/Asn142/Thr26, respectively [32,66].

#### 2.2.2. RdRp and Helicase

In the terms of virus replication, RdRp is important for SARS-CoV-2 genome replication/RNA transcription cycles [67,68]. In vitro, luteolin displays a potential inhibition activity against RdRp with IC_50_ value of 4.6 µM and demonstrates stable binding within the BRNA and BNTP binding pockets [69]. Moreover, luteolin interacts with the RdRp enzyme through hydrogen bonds with the amino acid residues Thr394, Arg457, Asn459, and Asn628, resulting in a binding energy of −7.5 kcal/mol [70].

Helicase utilizes ATP hydrolysis to facilitate the separation and rearrangement of nucleic acid duplexes, which are responsible for unwinding double-stranded DNA into single strands, thereby facilitating replication. Potential therapeutic agents, such as serine protease inhibitor and ACE2 blocker luteolin, may be investigated for its ability to mitigate SARS-CoV-2 infection by targeting viral components such as 3CL^pro^, PL^pro^, RdRp, and helicase [71].

## 3. Luteolin Regulates Host Receptor/Protease Activity to Inhibit Viral Invasion

**Figure 3:**
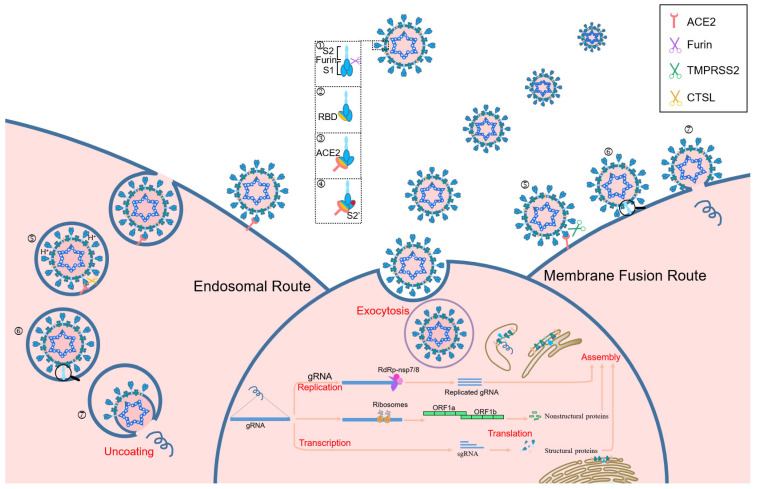
Structure of SARS-CoV-2 and infection cycles. Schematic representation of the principal entry routes SARS-CoV-2 uses for infection. When SARS-CoV-2 is released by the parental cell, S protein is cleaved by host furin, which facilitates faster binding to the functional receptor ACE2 and induces a conformational change which exposes the S2′ cleavage site. The presence or absence of TMPRSS2 dictates whether the virus enters through a fast membrane fusion or a slow endosomal route. In the absence of TMPRSS2, the virus is taken into an endosome where the pH will drop, activating cathepsin L. Cathepsin L cleaves S protein to initiate fusion to the endosomal membrane before release of viral RNA into the cytosol. In the presence of TMPRSS2, the S2′ site is cleaved and the virus can fuse directly to the cell membrane, allowing for a more rapid entry of the viral RNA into the cell. 1. S1/S2 cleavage by parental furin. 2. Open conformation exposing RBD. 3. Spike RBD binding to ACE2. 4. Conformation change and exposure of S2′. 5. Cleavage of TMPRSS2 or cathepsin L to expose the fusion domain. 6. Fusion. 7. Genomic RNA release.

During viral infection, the host cell proteases, cellular transmembrane protease serine 2 (TMPRSS2) and furin, cleave S protein into S1/S2 subunits, which is important for receptor identification and virus–cell fusion [72,73,74]. SARS-CoV-2 identifies host cell receptors, specifically ACE2 and CD147, by binding to the RBD located in the N-terminal domain of S1 subunit. This interaction facilitates the formation of a six-helical bundle between heptad repeats 1 and 2 in the S2 subunit, which subsequently induces conformational changes [75,76,77]. The S protein of SARS-CoV-2 promotes the entrance of the virus into host cells by binding S1 subunit to ACE2 cellular receptor, which facilitates viral attachment to the surface of infected cells [78]. Additionally, entry requires priming of the S protein by TMPRSS2, involving cleavage at both S1/S2 and S2′ site and enabling membrane fusion between virus and cells through the action of S2 subunit [72]. Consequently, a fusion pore is formed, allowing the transfer of viral RNA-associated nucleocapsid proteins from viral lumen into cytosol of host cell, thereby initiating the infection process (Figure 3) [79]. Without the presence of ACE2 or TMPRSS2, SARS-CoV-2 predominantly utilizes transmembrane glycoprotein CD147-mediated endocytosis and undergoes cleavage at the S2′ site by cathepsin L within the endolysosome [54,80,81].

Within host cells, SARS-CoV-2 makes use of the endogenic cellular machinery to transcribe and replicate viral RNA. After entering host cells, the genomic RNA (gRNA) undergoes translation by ribosomes to form polyprotein pp1a/pp1b and is then auto-cleaved by 3CL^pro^ and PL^pro^ to produce nsps, which triggers a cellular membrane rearrangement resulting in double-membrane vesicles that serve as anchor points for viral replication complexes [82]. Utilizing the gRNA as a template, SARS-CoV-2 replicase catalyzes the synthesis of full-length negative sense (−) gRNA, which is subsequently used for a template to generate a complementary positive sense (+) gRNA and a group of various sub-genomic RNAs, essential for coding the structural and accessory proteins of SARS-CoV-2, and culminating in virion assembly and release [83,84].

As previously noted, ACE2 receptor mediates the entrance of virus into human cells. Emerging data suggest that several cellular mediators and receptors might also promote the infection of host cells, such as CD147 and angiotensin II receptor type 2 [80,85,86]. Additionally, TMPRSS2 and furin, which exist in various endocytic compartments and cell membranes, exert a crucial role in S protein priming for efficient endocytosis and subsequent release of viral genome into infected host cells [87,88,89].

### 3.1. Downregulation of ACE2 Expression and Disruption of CD147-Mediated Endocytosis

The host cell surface ACE2 receptor exhibits high expression levels in the lung, which is key to the life cycle of SARS-CoV-2 [90]. The interaction between the virus and ACE2 receptor reveals that the SARS-CoV-2 utilizes anchoring residues Tyr453, Tyr500, and Tyr505 to achieve robust binding with His34, Arg393, and Lys353.

Among 42 bioactive compounds from *Cannabis sativa* L., luteolin is the best inhibitor against S binding pocket ACE2 through the analysis of molecular docking and molecular dynamic simulation [32,91]. By disrupting the interaction of several residues, including Gly496, Gln498, Tyr505, Leu455, Gln493, and Glu484 in the RBD of the S protein, as well as Lys353, Asp30, and Tyr83 in the ACE2 receptor, luteolin diminishes the affinity of the RBD for ACE2 and obstructs viral entry into host cells [31]. In addition, it has been reported that luteolin exhibits a higher affinity for the ACE2 receptor by forming hydrogen bonds with the amino acid residues Gln81, Gln101, and Asn194, with a binding energy of −6.0 kcal/mol [45].

CD147, a transmembrane glycoprotein, is a part of immunoglobulin superfamily expressed in lungs. Investigations reveal that CD147 provides an alternative route for virus infection and potentially mediates SARS-CoV-2 entry, especially in ACE2-deficient cell types [76,92]. CD147 can interact with the S protein and is able to penetrate host cell cytoplasm by endocytosis, which activates NLRP3 inflammasome that cleaves IL-1β and IL-18 in COVID-19 patients [92]. Isoorientin, a natural flavonoid substance of luteolin glycosides, inhibits cell migration by inhibiting expression/activity of CD147 receptor in human lung cancer cells [93].

### 3.2. Blockage of Viral Entry Pathways by Targeting Protease Activities

Besides cleavage at S2′ site mediated by TMPRSS2, furin and trypsin can process the cleavage at the S1/S2 site, as well as two cleavage sites in S1 region by cathepsin L (Figure 2B) [88,94]. The presence/absence of TMPRSS2 determines the mode of viral entry, either via a fast or slow membrane fusion/endosomal route. With the presence of TMPRSS2, cleavage occurs at S2′ site and enables direct fusion of the virus with host membrane, which accelerates the entry of viral RNA into the infected cells (Figure 3). Conversely, without TMPRSS2, SARS-CoV-2 is internalized into the endosome, which activates cathepsin L to cleave the S protein, thereby triggering fusion with the endosomal membrane and facilitating the release of viral RNA into the cytosol [88,95].

Research indicates that luteolin interacts with the TMPRSS2 protein through hydrogen bonding at the Lys390, Ser436, and Ser441, as well as hydrophobic interactions with Glu389, Asp435, Cys437, Gln438, Thr459, Ser460, Trp461, Gly464, Cys465, and Gly472 residues. The binding energy and inhibition constant for this interaction is −6.8 kcal/mol and 10.39 µM, respectively [45,96]. In detail, luteolin inhibits alpha-coronavirus infection dose-dependently, with IC50 values of 1.77 µM and 1.95 µM in TMPRSS2-expressing and non-expressing Huh-7 cells, respectively [97].

Studies from biochemical interrogation of furin show that luteolin may act on furin substrate complex to restrict the virus maturation [98], rendering the development of luteolin targeting host proteases an attractive clinical prospect [99].

Cathepsin L has been identified as a protease that cleaves the S protein within the endosomes in TMPRSS2-deficient cells and participates in membrane fusion activation [100,101]. The course of exosome biogenesis includes formation through cell membrane by endocytosis, along which cold-inducible RNA-binding protein is packaged. It has been found that luteolin can antagonize RNA-binding protein, which plays a role in inflammatory responses by inhibiting macrophage-mediated pathways [32].

## 4. Pathways Are Predicted with Luteolin Involved in SARS-CoV-2

Oxidative stress is a crucial factor that causes metabolic and physiological alterations and various diseases of the organism. Studies demonstrate that oxidative stress is closely linked to multiple pathological changes in COVID-19 patients, actively contributing to the exacerbation and progression of cytokine storm, coagulation disorders, and cellular hypoxia [102]. The interaction of the viral S protein with ACE2 leads to an excessive production of angiotensin II and activation of NADPH oxidase, which subsequently results in the enhancement of oxidative stress mechanisms and the release of inflammatory molecules [103]. As mentioned earlier, luteolin blocks SARS-CoV-2 viral entry by disrupting key residue interactions between the S protein’s RBD (Gly496, Gln498, Tyr505, etc.) and ACE2 (Lys353, Asp30, etc.), while also forming stabilizing hydrogen bonds with ACE2 residues (Gln81, Gln101, Asn194). Meanwhile, ROS can induce tissue damage, thrombosis, and red blood cell dysfunction, contributing to the severity of COVID-19 disease. The high ratio of neutrophils to lymphocytes observed in critically COVID-19 patients has been reported to be associated with excessive levels of ROS, which promotes a cascade of biological events that drive host pathological responses. Moreover, COVID-19 causes the death of infected cells, activation of the innate immune response, and secretion of inflammatory cytokines [104]. All of these processes are associated with oxidative stress, which makes an essential contribution to the pathogenesis of SARS-CoV-2 infection.

**Figure 4:**
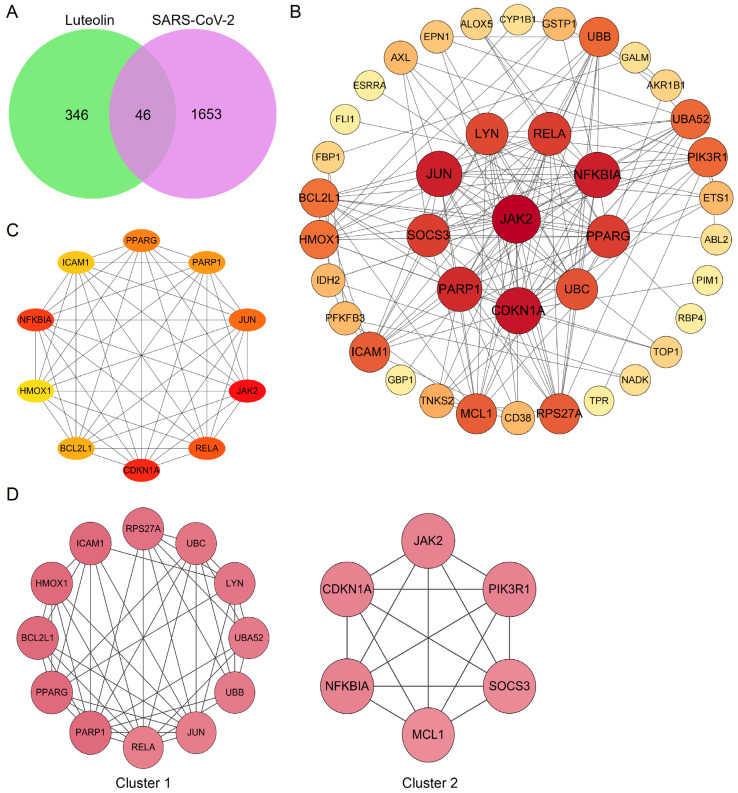
Bioinformatics analysis of overlapping genes. (**A**) Venn diagram of 46 overlapping genes between the predicted targets of luteolin and the targets associated with SARS-CoV-2. (**B**) PPI network of the 46 overlapping genes. The importance of each gene was analyzed by STRING. The higher the degree, the more crucial the gene. The degree of the outermost, middle, and smallest circle was 0–13, 14–20, and 22, respectively. The colors of nodes from reddish to yellowish were arranged in descending order on the basis of their degree values. (**C**) Cytohubba, the plug-in of Cytoscape, was used to analyze the top 10 hub gene network of target proteins by MCC algorithm. (**D**) Cluster of the 46 overlapping gene containing PPI network. Two clusters were identified. Cluster 1 had the highest score of 8.18, and PARP1 was identified as the seed gene. Cluster 2 had a score of 6, and CDKN1A was the seed gene.

**Figure 5:**
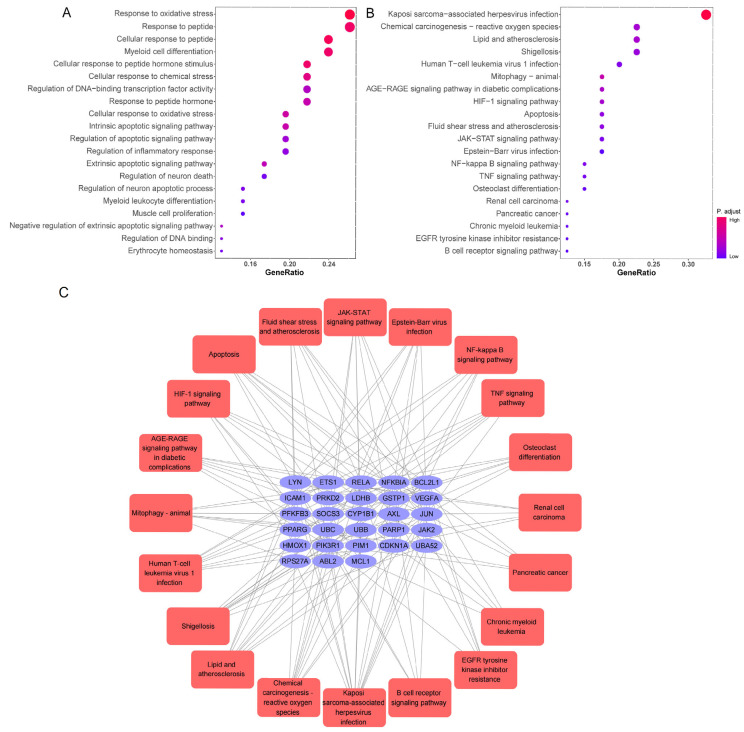
GO and KEGG analysis of 46 common genes. (**A**) Bar chart of top 20 enriched GO items of potential targets associated with the molecular function. (**B**) Bar chart of top 20 KEGG signaling pathways related to the effect of luteolin against SARS-CoV-2. The redder the bubble, the smaller the *p*-value; the larger the bubble, the greater the number of genes that participated in this pathway. (**C**) The critical signaling pathway target visualization network. PPI network of the top 20 KEGG signaling pathways and associated target genes. Red nodes represent top 20 KEGG pathways, and blue nodes indicated the genes that participated.

The herbal compound luteolin is posited to effectively inhibit inflammatory responses, thereby mitigating the ‘cytokine storm’ through downregulation of various inflammatory mediators, including IL-1β, IL-6, IL-17, TNF-α, MAPK family members, STAT3, etc. [105,106,107,108,109]. Network pharmacology quantifies the relationship between disease-related genes and drug targets, which facilitates drug repositioning [32]. The 392 active targets of luteolin from BATMAN, HERB, TCMSP, SwissTargetPrediction databases and 1699 targets associated with SARS-CoV-2 were collected using the network pharmacology analysis, which showed 46 common genes (Figure 4A). Meanwhile, a protein–protein interaction (PPI) network was constructed to illustrate the interactions among these gene targets (Figure 4B). The hub genes were identified by Cytoscape_3.7.2 plug-in Cytohubba, utilizing the Maximum Clique Centrality (MCC) algorithm, which assigns node ranks based on the extent of their participation in all maximal cliques across the network [110]. The top ten hub genes include JAK2, RELA, CDKN1A, BCL2L1, HMOX1, NFKBIA, ICAM1, PPARG, PPRP1, and JUN (Figure 4C). Three distinct clusters were obtained using the Molecular Complex Detection, among which Cluster 1 and 2 had higher scores, and PARP1 and CDKN1A were identified as the seed genes, respectively (Figure 4D). PARP1 demonstrated significant therapeutic potential in lung injury, especially COVID-19, and was implicated in regulating host responses to SARS-CoV-2 infection, serving as a potential marker for pulmonary inflammatory diseases. Molecular docking studies further substantiated that luteolin could bind to PARP1 [111,112]. Notably, the activity of luteolin was underscored by findings showing it increased cleaved PARP1 levels in a concentration-dependent manner [113]. Moreover, lung injury increased the gene expression of cell cycle inhibitor CDKN1A which reversed by luteolin in A549 cells, and resulted in G1 phase arrest [114,115,116]. Gene Ontology (GO) analysis and Kyoto Encyclopedia of Genes and Genomes (KEGG) enrichment analysis were then used to explore the potential signaling pathways. The findings from the molecular function enrichment analysis demonstrated that cellular response, apoptosis and inflammatory, were closely related to the pathological mechanisms of COVID-19 (Figure 5A). The KEGG pathway analysis highlighted key pathways associated with multiple ‘virus infections’, ‘HIF-1’, ‘JAK-STAT’, ‘NF-kappa B’, and ‘TNF’ (Figure 5B). A comprehensive visualization of the critical pathways is summarized in Figure 5C. Based on the predicted analyses in this study, we can speculate that luteolin has broad antiviral activities by targeting special proteins required for SARS-CoV-2 infection, through diverse mechanisms [45,117]. Possession of anti-oxidant and anti-inflammatory effects also creates potential possibilities for luteolin against SARS-CoV-2 [46]. The activation of the NF-κB signaling pathways leads to the production of ROS, an increased myeloperoxidase expression, as well as the expression of pro-inflammatory molecules and chemokines [104]. The pivotal molecular mechanisms of luteolin antagonizing COVID-19 may lie in the improvement of vascular circulation through inhibiting the vascular inflammation triggered by NF-κB and TNF-α, and has a n important beneficial effect on improving unusual angiogenesis and vascular leakage during COVID-19 [118,119].

## 5. Conclusions and Future Perspective

With the development of network pharmacology-based artificial intelligence technology and ongoing study for COVID-19 treatment, it is likely to lead to the identification and clinical application of an increasing array of natural antiviral nature compounds. The integration of herbal therapies that exhibit complementary and synergistic effects to impede or obstruct virus–host interactions is poised to serve as a formidable strategy in combating COVID-19.

This study investigates the mechanisms by which Luteolin targets SARS-CoV-2, revealing its broad-spectrum antiviral properties that operate through multiple pathways. Analysis of the targets/action mechanism of luteolin indicates a wide range of targets on both SARS-CoV-2 and human cell receptors (Table 2). Potential therapies of luteolin against SARS-CoV-2 infection can be classified into three categories. In the first case, the therapies can target structural and functional proteins/enzymes or the genetic material of the SARS-CoV-2 to directly inhibit viral replication by obstructing RNA synthesis, replication, or self-assembly. In the second instance, therapies can inhibit/block the SARS-CoV-2 from binding to host receptors, impeding virus entry and spreading among cells. Finally, the remedy can be accessed by anti-oxidant and anti-inflammatory effects to suppress inflammatory responses, reduce cellular apoptosis, enhance vascular circulation, and diminish vascular leakage.

The findings of this study indicate that luteolin has considerable potential as an anti-SARS-CoV-2 agent via direct (antiviral activity) and indirect (anti-oxidant and anti-inflammatory) mechanisms. Nevertheless, further in vivo and in vitro studies are required to substantiate its efficacy and safety. Choosing the appropriate route of administration and preparing the drug into applicable formulations can improve bioavailability. We are expecting more studies to explore the medicinal value of luteolin and to develop flavonoid-based antiviral drugs. Further post-drug discovery research would provide a deeper perspective on the efficacy and safety of nature compounds and isolated substances for sole use, herb–herb, or drug–herb combinations. Luteolin, a natural antiviral compound, will provide novel insights and methodologies for the management of SARS-CoV-2 infection and emerge as a highly efficacious intervention in the global effort to combat the ongoing pandemic.

## Figures and Tables

**Table 1:** Physicochemical properties of luteolin.

Property	Index	Druggability
Molecular weight	286.24	<500
Melting point	329.5 ± 1.0 °C	>160 °C, easy to dry, simple process
Boiling point	616.1 ± 55.0 °C	
Density	1.65 ± 0.06 g/cm^3^	>1 g/cm^3^, easy crystallization
pKa	6.50 ± 0.40	Non-dissociated type, easy to absorb
Solubility	Soluble in ethanol and diethyl ether, and slightly soluble in hot water	Poor lipophilicity and hydrophilicity

**Table 2:** Molecular docking sites of luteolin against SARS-CoV-2 and host receptors.

Target Protein	Hydrogen Bonds	Hydrophobic and Van Der Waal Interactions	Binding Energy (kcal/mol)	Reference
S	Asn343, Asp364	Gln804, Asn801	−5.1	[44,45]
N	Asn76	Ser79, His146, Ile147, Trp53, Ala156, Ile158	−7.5	[53]
3CL^pro^	Gln189, Leu4, Asn142, Thr26	Met49, Val3	−8.1	[32,66]
RdRp	Thr394, Arg457, Asn459, Asn628		−7.5	[70]
ACE2	Gln81, Gln101, Asn194		−6.0	[45]
S and ACE2 complex	sp30, Tyr83, Lys353, Gln493, Gly496, Gln498	Tyr505, Leu455	−6.6	[31]
TMPRSS2	Lys390, Ser436, Ser441	Glu389, Asp435, Cys437, Gln438, Thr459, Ser460, Trp461, Gly464, Cys465, Gly472	−6.8	[45,96]

## Data Availability

No new data were created or analyzed in this study. Data sharing is not applicable to this article.

## Abbreviations

The following abbreviations are used in this manuscript:

3CL^pro^	3-chymotrypsin-like protease
ACE2	Angiotensin-converting enzyme 2
ALI	Acute lung injury
ARDS	Acute respiratory distress syndrome
COVID-19	Coronavirus disease 2019
N	Nucleocapsid
Nsp	Non-structural protein
NTD	N-terminal domain
ORFs	Open reading frames
PL^pro^	Papain-like protease
RdRp	RNA-dependent RNA polymerase
S	Spike
SARS-CoV-2	Severe acute respiratory syndrome coronavirus 2
TMPRSS2	Transmembrane protease serine 2
